# Successful apexification with resolution of the periapical lesion using mineral trioxide aggregate and demineralized freeze-dried bone allograft

**DOI:** 10.4103/0972-0707.66723

**Published:** 2010

**Authors:** Naveen Chhabra, Kiran P Singbal, Sharad Kamat

**Affiliations:** Department of Conservative Dentistry and Endodontics, Institute of Dental Sciences, Bareilly, India; 1 Department of Conservative Dentistry and endodontics, Vyas Dental College, Jodhpur, India; 2 Department of Conservative Dentistry and endodontics, PMNM Dental college and hospital, Bagalkot, India

**Keywords:** Allograft, apexification, calcium hydroxide, mineral trioxide aggregate, root canal therapy

## Abstract

Immature teeth with necrotic pulp and large periapical lesion are difficult to treat via conventional endodontic therapy. The role of materials such as calcium hydroxide and mineral trioxide aggregate in apexification is indispensable. This case report presents the successful healing and apexification with combined use of white mineral trioxide aggregate and demineralized freeze-dried bone allograft.

## INTRODUCTION

Complete asepsis and three-dimensional obturation of the root canal system are essential for long-term endodontic success. In certain cases such as immature teeth, the absence of natural apical constriction creates a challenge. Therefore, one of the aims of endodontic treatment is to produce an apical barrier or stop, against which one can place a root canal filling material avoiding overextrusion. This technique is termed apexification.

Clinicians have tried several materials to form apical barrier in the past. These include: calcium hydroxide paste, calcium hydroxide powder; mixed with different vehicles,[1–3] tricalcium phosphate,[4] collagen calcium phosphate,[5] osteogenic protein-1, bone growth factor and oxidized cellulose.[6] proplast, (a polytetrafluor-ethylene and carbon felt-like porous material),[7] barium hydroxide,[8] true bovine bone ceramics,[9] and dentin chips. Antibacterial such as paste of metronidazole, ciprofloxacin, and cefaclor has effectively encouraged apexification.[10] Deliberate over instrumentation of the periapical area to produce a blood clot that will induce apical closure has also been described.[11]

Mineral trioxide aggregate (MTA) was developed at Loma Linda University for use as a root-end filling material.[12] MTA has shown potential outcome in carrying out apexification of immature permanent teeth.[13,14] Apexification using MTA has several advantages such as it neither gets resorbed, nor weakens the root canal dentin, and also sets in the wet environment. Satisfactory compaction of obturating material is achievable as MTA on setting provides a sound and hard apical barrier.

Bio-resorbable demineralized bone matrix (DMBM) is the protein component of bone and is widely used in various clinical conditions such as periodontal defects and oral and maxillofacial bone defects. Periodontal defects grafted with demineralized bone matrix allograft showed histologic evidence of regeneration of new bone and periodontium.[15] Considering the osteoconductive potential and proven success of demineralized bone matrix allograft in the management of periodontal defects, it provides an excellent alternative for use in management of large periapical radiolucency.

The apical matrix of some resorbable and biocompatible material is essential to control extrusion of MTA. “Modified matrix concept” for repair of perforation utilized resorbable collagen as a matrix followed by condensation of MTA.[16] Considering the biocompatible nature of bio-resorbable demineralized bone matrix, it could be the material of choice in such cases.

Therefore, present case report highlights the nonsurgical management of symptomatic tooth with blunderbuss canal and large periapical radiolucency using bio-resorbable demineralized bone matrix and MTA.

## CASE REPORT

**Figure 1:**
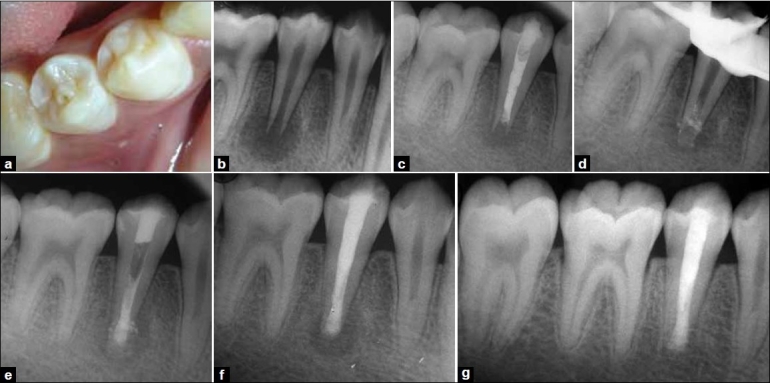
(a) Right mandibular second premolar showing the presence of dense evaginatus, (b) Preoperative radiograph of right mandibular second premolar showing deep pit communicating with pulp space along with blunderbuss canal and large periapical radiolucency, (c) 15 days recall radiograph after the placement of calcium hydroxide and iodoform paste, (d) Apical matrix of decalcified freeze dried bone allograft, (e) Apical plug of MTA created over the apical matrix, (f) 3 months followup radiograph showing reduction in the size of the periapical lesion, (g) 2 years follow-up radiograph representing complete resolution of the periapical lesion

A 16-year-old male patient of south Indian origin reported to the department of conservative dentistry and endodontics, PMNM Dental College and Hospital, Bagalkot, Karnataka, India, with the complain of pain in right mandibular posterior teeth since 3 weeks. Careful intraoral examination revealed sinus opening in relation to the right lower second premolar. Hard tissue examination revealed the presence of “dens evaginatus” and a deep pit in right mandibular second premolar [Figure 1a]. Concerned tooth did not respond to electric pulp testing. Radiographic examination revealed deep pit communicating with the pulp space, presence of blunderbuss canal, and large periapical radiolucency with respect to right mandibular second premolar [Figure 1b]. There were two treatment options- either surgical removal of the periapical lesion followed by retrograde filling or nonsurgical endodontic treatment consisting of routine endodontic therapy and apexification using mineral trioxide aggregate. Nonsurgical treatment was opted considering the age and amount of trauma expected during surgical treatment. Local anesthesia was not required as tooth was nonvital. Access was prepared under rubber dam isolation. Pus exuded through the canal immediately after access preparation. Canal was irrigated using lukewarm normal saline to assist in exudation. Access preparation was left open until exudate stopped coming out. This followed thorough biomechanical preparation, involving circumferential filling with a size 80 K file (Dentsply, India) to remove any debris or necrotic dentin and root canal irrigation with 1.25% sodium hypochlorite solution. Thereafter, calcium hydroxide and iodoform combination (Metapex™, META Biomed Co. Ltd., Korea) was placed in canal and patient was recalled after 15 days [Figure 1c]. Recall appointment showed the healing sinus and patient was asymptomatic. The medicament was removed from the canal followed by irrigation with 1.25% sodium hypochlorite. After confirming dryness of canal, the apical matrix/barrier was created via pushing decalcified freeze-dried bone allograft (Osseograft ™, Advanced Biotech Products (P) LTD, India) through the canal using finger pluggers (Dentsply, India) and packing it in periapical area [Figure 1d]. This was followed by a placement of 5 mm apical plug of white mineral trioxide aggregate (PROROOT MTA ™ Dentsply, India) using a finger plugger. Keeping moist cotton over the canal orifice achieved complete setting of MTA, which was followed by closure of access preparation using an interim restorative material (Cavit G™ 3M ESPE, India) [Figure 1e]. The patient was asymptomatic at 1-week recall visit. Therefore, remaining canal was obturated using resin-based endodontic sealer (AH 26, Dentsply India) and thermoplasticized gutta percha (Obtura II, J. Morita Corporation, Japan). The 6-month follow-up radiograph of the patient showed reduction in the size of the periapical lesion [Figure 1f]. At 2-year recall, the patient was completely asymptomatic and intraoral periapical radiograph of the same tooth revealed complete resolution of the periapical lesion [Figure 1g].

## DISCUSSION

Despite the higher success rate of apical barrier formation using calcium hydroxide, long-term follow-up is essential. Problems such as failure to control infection, recurrence of infection, and cervical root fracture may occur.[17,18] Apexification using mineral trioxide aggregate provides an alternative treatment modality in immature pulpless teeth. Apexification with MTA requires significantly less time.[19] Mineral trioxide aggregate as an apexification material represents a contemporary version of the primary monoblock. Apatite-like interfacial deposits form during maturation of MTA result in filling up of gaps induced during the material shrinkage phase and improve the frictional resistance of MTA to the root canal walls. The formation of nonbonding, gap-filling apatite deposits probably also accounts for the seal of MTA in orthograde obturations and perforation repair.[20]

MTA has superior biocompatibility and sealing ability and is less cytotoxic than other materials currently used in pulpal therapy.[21] The 5-mm barrier is significantly stronger and shows less microleakage as compared to the 2-mm barrier of MTA.[22]

Apexification using MTA lessens the treatment time between the patient’s first appointment and the final restoration. The importance of this approach lies in the expedient cleaning and shaping of the root canal, followed by apical seal with a material that favors regeneration. In addition, there is reduced potential for fracture of immature teeth with thin roots, because of immediate placement of bonded core within the root canal.[23]

In the present case, combination of calcium hydroxide and iodoform was used as intracanal medicament for 15 days to make the canal dry and free from infection. Use of calcium hydroxide for such a short term does not adversely affect the fracture resistance of the tooth.[24]

The demineralized bone matrix acts as an osteoconductive and possibly as an osteoinductive material.[25,26] Hence, probably, allograft material could have promoted the healing in the present case. However, only objective of using bone graft material in this case was to produce an apical barrier. Use of the apical matrix of decalcified freeze-dried bone allograft material in the present case provided excellent compaction of mineral trioxide aggregate, while minimizing extrusion of mineral trioxide aggregate in the periapical area.

Decalcified freeze-dried bone allograft material has been extensively used in the management of extensive periodontal defects. Its use results in significant probing depth reduction, clinical attachment gain, and bone fill. Definite evidence exists that sites grafted with DFDBA heal with regeneration of periodontium.[27,28] Follow-up radiographs also showed the excellent healing with resolution of periapical pathology. Recent literature reported the successful use of combination of hydroxyapatite and platelet rich plasma in surgical management of the large periapical lesion with open apex.[29] Combination of hydroxyapatite and platelet-rich plasma or demineralized bone matrix and platelet-rich plasma can also be tried using the method as stated in the present case in further case studies.

‘Dens evaginatus’ or ‘evaginated odontoma’ is a developmental anomaly that occurs more often in mandibular premolars;[30] however, it can also affect other teeth, including supernumerary teeth.[31] It is the result of an abnormal proliferation of the inner enamel epithelium into the stellate reticulum of the enamel organ.[32] The resulting tubercle contains a core of dentin surrounding a pulpal extension, which may be narrow, wide, constricted, an isolated horn, or not present at all.[32] The prevalence of Dens evaginatus is between 1% and 4%. It occurs most commonly in people in the Mongoloid racial group, which includes the Paleo-Asiatics (Indians of North, Central and South America and Eskimos), the Neo-Asiatics (Chinese, Thais and Japanese), and the Indonesian-Malays (Filipinos).[32] In this case, probably invasion of salivary fluids and microorganisms through the enamel of the occlusal table caused the damage. Early detection of dens evaginatus is important—it may be possible to fissure seal over the defect using bonded restorative, or cut the defect away and perform an MTA pulpotomy.

This case report presents a novel approach to achieve single visit apexification of the cases with open apex and large periapical lesion. Present case also stresses the early detection and treatment of ‘dens evaginatus’ which if undetected can cause undue damage.
